# Supplementary material for: Korean Red Ginseng Ameliorates Allergic Asthma through Reduction of Lung Inflammation and Oxidation
Source: Antioxidants (Basel). 2022 Jul 22;11(8):1422. doi: 10.3390/antiox11081422 (PMC9331112; doi:10.3390/antiox11081422)
Supplement: Supplementary file 1 [file antioxidants-11-01422-s001.zip › antioxidants-1792061-supplementary.pdf]

antioxidants

Supplementary Materials

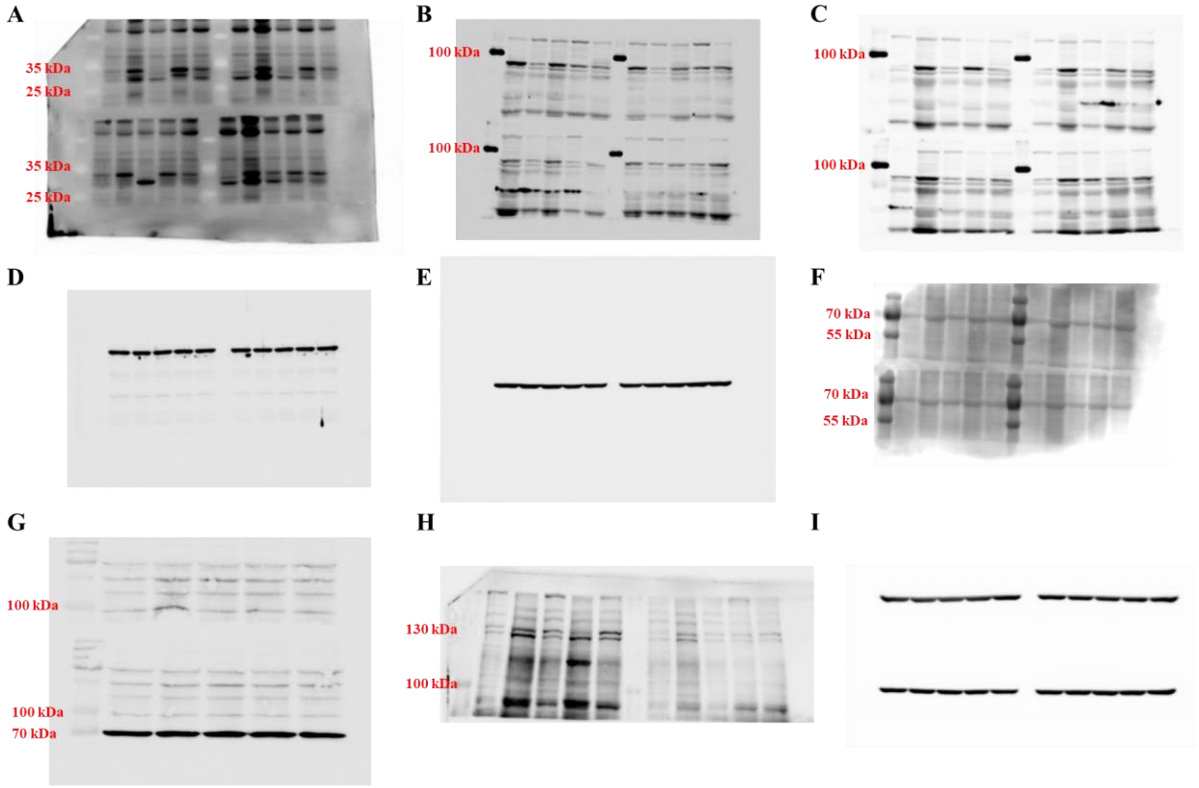

Figure S1. Original band image of Western blot with protein marker. HO-1 (A), cytoplasmic Nrf2 (B), nuclear Nrf2 (C), α-tubulin (D), lamin B1 (E), p-p65 (F), t-p65 (G), iNOS (H), and β-actin (I).
